# Ovarian Follicles Rescued 3 Days after Cyclophosphamide Treatment in Adolescent Mice: An Experimental Study Aiming at Maximizing Methods for Fertility Preservation through In Vitro Follicle Culture

**DOI:** 10.3390/ijms20246190

**Published:** 2019-12-07

**Authors:** Amandine Anastácio, Max Waterstone, Xia Hao, Catherine Poirot, Kenny A. Rodriguez-Wallberg

**Affiliations:** 1 Department of Oncology-Pathology, Karolinska Institutet, SE-171 76 Stockholm, Sweden; amandine.anastacio@ki.se (A.A.); waterstm@tcd.ie (M.W.); xia.hao@ki.se (X.H.); 2 Laboratory of Translational Fertility Preservation, BioClinicum, SE-171 64 Stockholm, Sweden; 3 Trinity Translational Medicine Institute, St James Hospital, D08W9RT Dublin, Ireland; 4 Faculty of Medicine, Sorbonne Université, 75005 Paris, France; catherine.poirot@aphp.fr; 5 Service d’Hématologie, Unité AJA, Hôpital Saint Louis, 75010 Paris, France; 6 Department of Reproductive Medicine, Division of Gynecology and Reproduction, Karolinska University Hospital, SE-141 86 Stockholm, Sweden

**Keywords:** cancer therapy, cyclophosphamide, gonadotoxicity, ovarian follicles in vitro culture, randomized controlled study, experimental model, mice

## Abstract

There is currently a lack of knowledge about the feasibility of performing procedures for fertility preservation after chemotherapy treatment has been initiated. In this experimental controlled study using adolescent mice, we aimed to investigate if the chance of rescuing and growing in vitro secondary follicles (SeF) would be affected three days after a single injection of cyclophosphamide (CPA). The main outcomes included were: (1) The number of SeF with good morphologic quality obtained per ovary 3 days after CPA injection, (2) SeF development in culture, (3) small follicle density (SFD) on histology, and (4) apoptosis markers, including terminal deoxynucleotidyl transferase dUTP nick end-labelling (TUNEL), mRNA expression, and distribution of p 53 upregulated modulator of apoptosis (*Puma*) and phosphatase and tensin homolog (*Pten*). We found a 60% reduction of SeF obtained per ovary in all CPA-treated groups vs. controls. However, in vitro survival rates at culture day 12 and antrum formation were similar among all groups. On histology, SFD was only significantly reduced in the high CPA dose group. Apoptotic cells were mainly found in large growing follicles of CPA groups. Our study indicates the feasibility of SeF isolation and in vitro follicle culture 3 days following CPA treatment and a still preserved SFD, particularly following a low-dose CPA treatment.

## 1. Introduction

Survival following cancer diagnosis has significantly increased [1]. This has led to a focus on the impact that cancer treatments have on the quality of life of cancer survivors. Commonly used alkylating drugs, such as cyclophosphamide (CPA), are highly gonadotoxic and a significant proportion of female cancer survivors develop premature ovarian failure and subsequent infertility [2,3]. In clinical medicine, options for fertility preservation have been developed and an increasing demand for fertility preservation procedures has been reported [4], with a growing number of research reports in this field [5,6]. There is, however, a need to understand the molecular mechanisms by which chemotherapeutic agents damage the ovary and to examine the effect that such treatments might have on the clinical implementation of fertility preservation techniques [7].

Established available options to preserve female fertility include gamete and embryo cryopreservation. To cryopreserve mature oocytes and/or embryos, treatment with hormonal stimulation for approximately 2 weeks is required. In situations where the initiation of cancer treatments is too urgent to delay, stimulation is contraindicated, or the patients are too young, the only option is to cryopreserve pieces of ovarian tissue. To date, the thawed tissue has had to be re-transplanted to regain functionality and to allow fertility restoration [8]. Follicular in vitro growth is also a promising method that has been proposed as a method for fertility restoration in the future, and entails the growth of early stage follicles to antral stage, whereupon ovulation can be induced using a human chorionic gonadotropin (hCG) trigger [9,10,11]. This would allow the growth of small immature follicles to a mature stage, thereby producing mature oocytes, an approach more suitable for cases with precluded re-transplantation due to an inherent risk of malignant cell invasion within the ovarian tissue. It is important to acknowledge that this technique has not yet been applied clinically, and to acknowledge its distinction from in vitro maturation (IVM), whereby immature germinal vesicle stage oocytes are obtained from antral follicles and attempts are made to mature them to metaphase II stage oocytes [12].

Clinical guidelines recommend the application of fertility preservation methods before cancer treatment initiation [8,13,14]. However, some patients are strongly advised not to delay treatment initiation, meaning that the effects of chemotherapy on their chances of obtaining a competent and mature oocyte after undertaking such an invasive procedure are of great interest. To date, little data are available on the effect that systemic chemotherapy administration might have on the development in vitro of ex vivo cultured secondary follicles, or the quality of the oocytes that they produce [9]. 

In this study, using adolescent mice, we aimed to investigate the effect of a recent CPA treatment in the feasibility of isolation and culturing of secondary follicles (SeF) with good morphologic quality and their development in vitro. We also wished to investigate any variations in the mice ovarian reserve estimating the small follicle density (SFD) on histological sections, and molecular markers of apoptosis, including TUNEL, *Puma*, and *Pten* mRNA expression and distribution. The culture method used in this study has been well established in previous reports and it allows daily observation of morphological characteristics to evaluate correct development of the follicles [15,16,17]. Using this culture system, we have previously reported on the establishment of the proteome of mouse follicles grown in culture at three developmental stages [17].

The approach of follicle rescue strategy is applied in this study as a possible future clinical strategy for fertility preservation, in particular for patients where ovarian transplantation may be precluded due to the risk of reintroducing malignant cells [10,11]. This study also aims to advance the collective knowledge of the oncofertility community on the molecular mechanisms by which chemotherapeutic agents produce their gonadotoxicity, furthering clinical and scientific aims alike.

## 2. Results

### 2.1. Follicle Isolation 3 Days after CPA-Injection and In Vitro Culture Outcomes

In this study, the mice were distributed in three groups according to the dose of CPA injected (50; 75; and 100 mg/kg), and a fourth group without CPA treatment was designated as a control.

**Figure 1:**
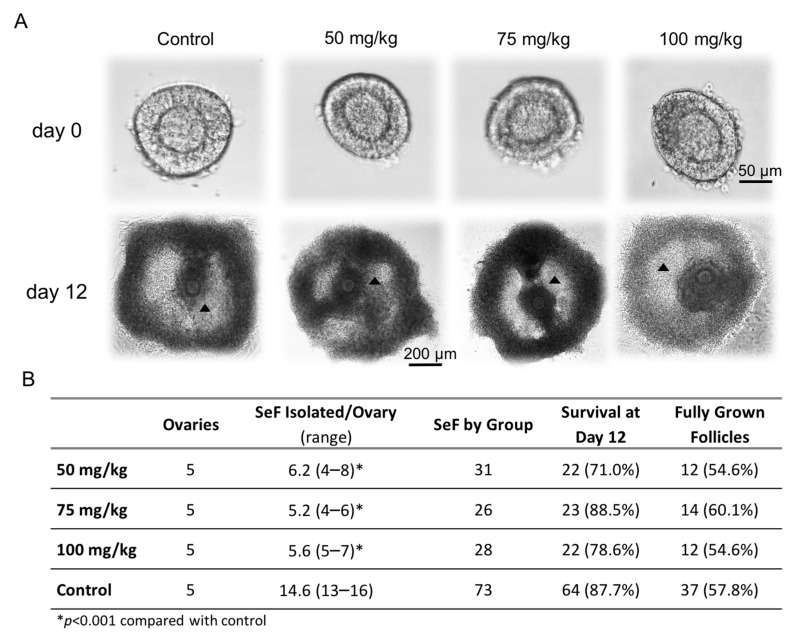
Isolated follicles fulfilling the criteria for culture in each of the groups. In this study, the right mouse ovary was used to isolate secondary follicles (SeF) for culture. The morphological characteristics of the selected follicles to be put in culture at day 0 can be observed in the upper panel (**A**), as well as the morphological aspect at culture day 12, which represents fully grown follicles (black triangles indicate the antrum-like cavity). The table in the lower panel (**B**) summarizes the number of SeF isolated per ovary fulfilling the criteria and thus put into culture (SeF isolated/ovary), the total number of follicles cultured (SeF by group), and the number of follicles that survived and were fully grown on culture day 12 by group. The percentage of survival (follicles with ≥200 µm and a visible round oocyte) was calculated as the proportion of follicles that survived from all follicles that were put into culture. The percentage of fully grown follicles in each group (≥450 µm and antrum-like cavity) was calculated by follicles that were considered to survive the 12 days of culture.

Figure 1A illustrates the required morphology and size of the SeF selected for culture, according to the protocol established by Cortvrindt et al. [15], three days after the CPA treatment. On the day of isolation, day 0, SeF fulfilling the morphologic criteria for culture were found in all groups. A mean of 14.6 SeF per ovary could be isolated and cultured in the control group, in contrast to 4 to 8 SeF obtained per ovary in the CPA-treated groups (*p* < 0.001 for all, Figure 1B). The reduction was approximately 60% in all CPA-treated groups vs. control. In the CPA-treated groups, the highest range (4–8 follicles) and average (6.2 SeF/ovary) were observed in the groups treated with the lowest dose of CPA (50 mg/kg). Interestingly, however, the likelihood of SeF isolation did not differ between the three different groups treated with CPA (Figure 1B), but the number of SeF fulfilling the morphologic criteria for culture was reduced in the CPA-treated mice. 

During culture, follicles obtained from CPA-treated mice, irrespective of dose, exhibited similar morphology and grew similarly to controls with antrum-like cavity formation in similar proportions (Figure 1A). The follicles that reached ≥200 μm on day 12 of culture were included in the survival ratio. Survival ratios did not differ significantly between the groups, ranging between 71% and 88.5% (Figure 1B). Of these, 55% or more were classified as fully grown follicles, i.e., reaching at least 450 μm in diameter and presenting an antrum-like cavity, independent of the CPA dose received (Figure 1B).

### 2.2. Follicle Growth Dynamics in Culture

**Figure 2:**
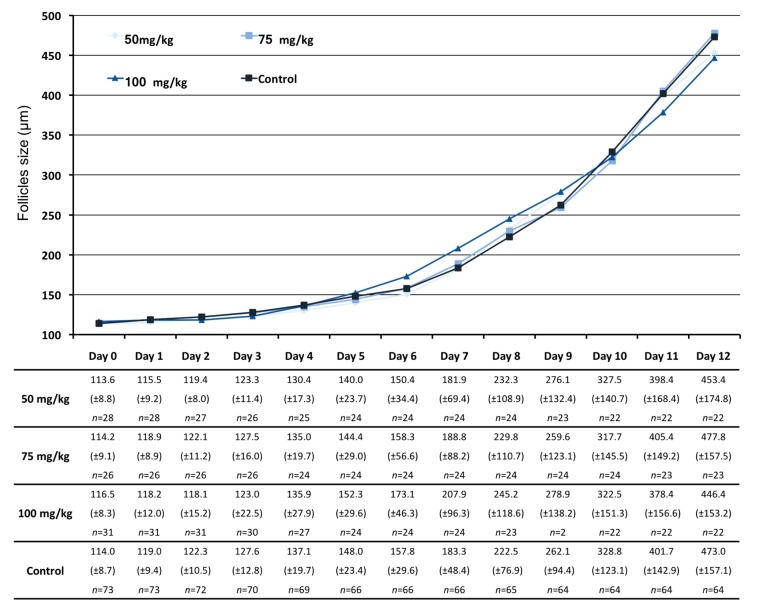
Follicular growth dynamics throughout culture. The curves were obtained using the mean of follicle diameters (µm) at each day of culture, giving the general dynamics of the follicular growth in each group. Only follicles with healthy appearance were measured and follicles that degenerated were discarded. The table indicates the mean follicle diameter (µm) by group at each day of culture with the standard deviation between parentheses and the number of follicles still alive at each day of culture.

Growth curves and follicular diameter were similar among all groups during culture. Follicular diameter increased slowly until day 6, approximately 2 to 10 µm/day, in all groups. From day 6, when we observed the rupture of the external membrane and the granulosa cells spread, as expected, the follicular diameter greatly increased in all groups, without differences between treatments. Although the follicles treated with the highest dose of CPA (100 mg/kg) grew apparently larger between day 6 till day 9 and presented with a largest average size in the middle culture days, this difference in growth was not statistically significant and the follicles reduced their growth thereafter, achieving a final smallest final average size on day 12 below 450 µm of diameter (±153.2) (Figure 2). 

### 2.3. Ovarian Reserve Estimated by Small Follicle Density (SFD) on Histology

**Figure 3:**
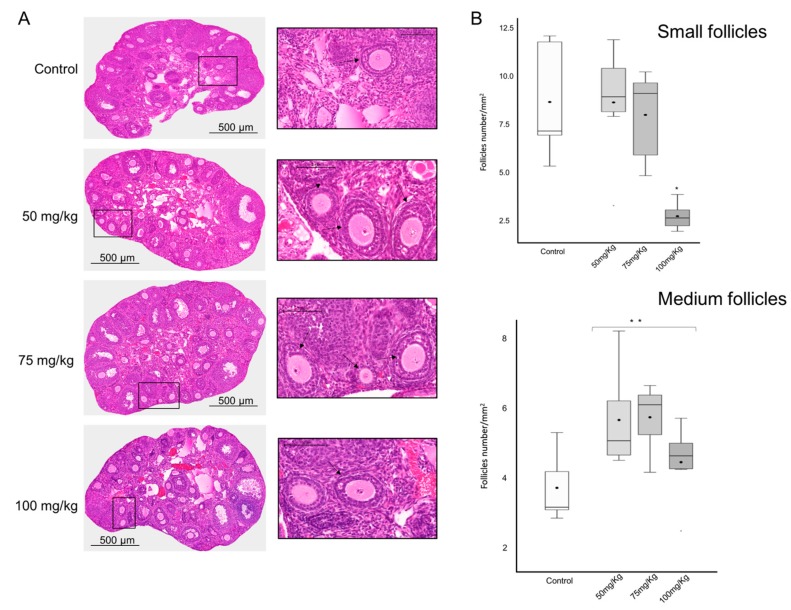
Follicle count and histological analysis. (**A**) H&E stained sections of each group illustrating in the box (×20) the follicles with the characteristics to be isolated and put in culture. Black arrows showing medium follicles and whitehead arrows showing small follicles. (**B**) Histogram of the mean density of small and medium follicles counted in the H&E stained sections of the different groups. * *p* < 0.001; ** *p* < 0.01.

In the hematoxylin and eosin (H&E) stained sections tissue morphology was similar among all groups (Figure 3A) without any abnormal fibrotic tissue observed. Follicles of all sizes (small, medium, and large) according to Pedersen and Peters’ classification [18] were observed in all groups, and the overall density and observed area was similar among the groups (data not shown). The small follicle density (SFD) was estimated by dividing the total number of small follicles per area observed for each ovary.

The SFD average was similar between the lower CPA dose groups (50 mg/kg: 8.7 (±3); 75 mg/kg: 8.1 (±2.5) small follicles/mm^2^); and the control (8.7 (±2.9) small follicles/mm^2^). However, a significant SFD reduction of 67% was found in the group treated with the highest CPA dose vs. the control, with an SFD average of 2.8 (±0.7) small follicles counted (*p* < 0.001) (Figure 3B). 

The medium follicle density, which includes growing follicles, with one to several layers of granulosa cells, was higher in the groups treated with CPA (*p* < 0.01). Thus, in the groups treated with the increasing doses of CPA, an average of 5.7 (±3.5), 5.8 (±3.2), and 4.5 (±2.6) medium follicles/mm^2^ were counted whilst 3.7 (±2.3) medium follicles were presented in the control group (Figure 3B).

### 2.4. Apoptosis Assessment 

**Figure 4:**
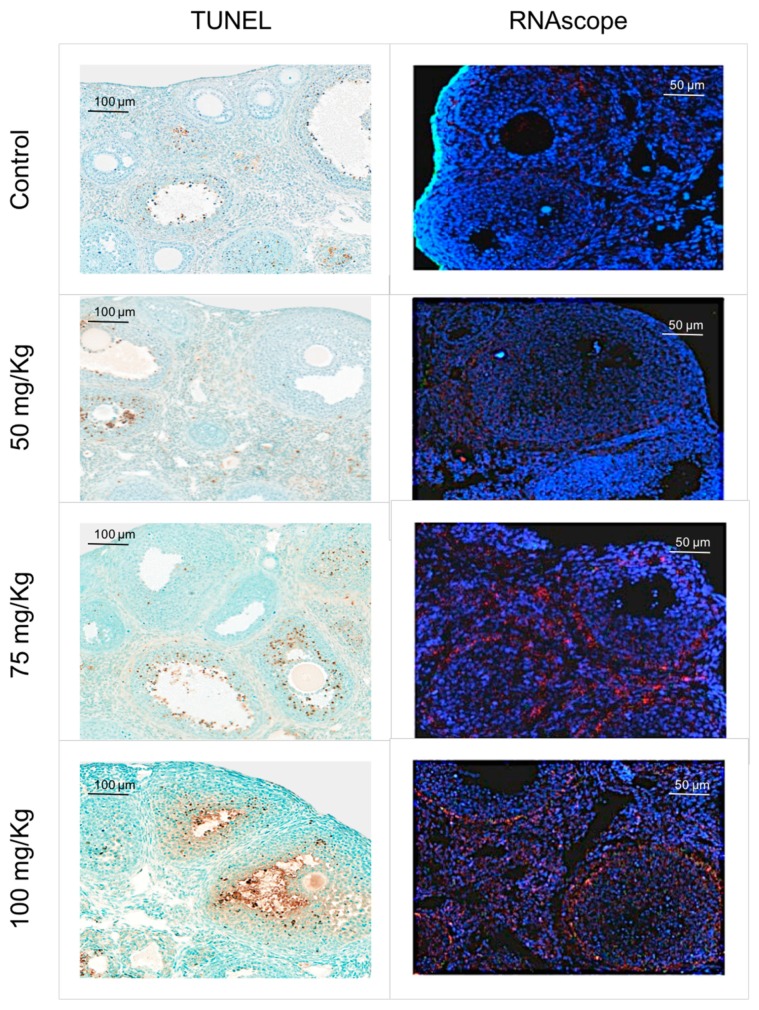
TUNEL and RNAscope. Results observed for the TUNEL and RNAscope assay. In the TUNEL assay (×20), the brown cells are positive for apoptosis. In the RNAscope (×40), *Pten* mRNA is represented by dots of red signal and *Puma* mRNA is represented by green.

TUNEL staining was performed in three ovarian sections from each treated group and two sections of the control group to assess in situ apoptosis. A qualitative analysis was carried out and the major results are displayed in a comparative grid (Figure 4). TUNEL-positive cells were mostly found in the granulosa cells of large follicles while little or no apoptotic staining was observed in the smaller follicles. The group treated with 100 mg/kg CPA exhibited a higher degree of apoptosis. 

### 2.5. Puma and Pten mRNA Distribution

A qualitative analysis was carried out in two sections from each group for RNAscope and the results are displayed in a comparative grid (Figure 4). Qualitatively, differences were observed between samples from mice treated with CPA, and those not treated with CPA, with those exposed demonstrating a high expression of *Pten* mRNA in a perifollicular pattern. This pattern tended to be located to theca cells in the groups treated with higher doses of CPA, although some stromal expression was also encountered across all samples. As such, it appears that CPA administration results in upregulation of *Pten* gene expression in ovarian theca cells. In contrast to this, the control and the group treated with the lowest dose of CPA demonstrated a pan-ovarian pattern of *Pten* expression.

*Puma* mRNA distribution analysis resulted in poor detection of fluorescence observed in the green channel (Figure 4). Yet, of all four groups, the highest expression was seen in ovaries treated with the high dose of CPA, 100 mg/kg. This expression was predominantly follicular and demonstrated a high degree of theca cells co-localization (Figure 4). 

## 3. Discussion

Clinical guidelines recommend fertility preservation prior to the initiation of cancer treatment [8,13,19]. However, some diseases require prompt initiation of the treatment and many patients may request undergoing fertility preservation after chemotherapy [13,19,20]. In this study, we wanted to investigate if it would be possible to attempt SeF rescue as a strategy for fertility preservation using in vitro growth and if the recent treatment with alkylating drugs in vivo would affect the growth and development ex-vivo of such obtained follicles. To our knowledge, no such previous investigation has been reported. 

Cyclophosphamide is a common alkylating drug used for cancer treatment. Its high gonadotoxic potential (impacting both dormant primordial follicles and growing follicles in vivo) has been demonstrated both in the animal/cell lines models and clinically [21,22,23,24]. This gonadotoxicity has been shown to be dose dependent [23,24,25], with a reduction of the ovarian reserve observable one week after the treatment, even at low doses (similar to the ones administered in our study) [24]. In this study, the ovaries were obtained 72 h after the CPA treatment. Although the number of secondary follicles isolated per ovary fulfilling the criteria to be put into culture was reduced in CPA-treated ovaries, no statistically significant differences were observed in follicle growth among the groups. A detailed description of the follicle size during culture is, however, hereby presented, as similar studies to ours using secondary follicles isolated three days after a CPA injection and grown in vitro have not been previously reported. No differences were observed in the ovarian reserve of mice treated with the lower doses of CPA compared to controls. Our data thus indicate that the damage induced by CPA on the ovarian follicular reserve may require a longer interval post-treatment to take effect, since previous studies have found a depleted ovarian reserve one week after CPA treatment using similar doses to the ones used in our study [24]. Notably, at the highest CPA dose, we already observed a reduction of more than 65% SFD in this study, contrasting with previous findings where no differences were observed for primordial follicles counted 3 days after they treated the animals with 120 mg/kg CPA [26]. Nevertheless, Chen et al. [26] provided scarce information about how follicular counting was performed and presented.

The finding that a population of growing follicles that was higher in all groups treated with CPA vs. control (*p* < 0.01) suggests that the follicular growth was accelerated by CPA treatment; a hypothesis previously cited to explain the “burnout” of the follicular pool following cancer treatment [27,28]. However, in this study, we did not observe a depletion of the ovarian reserve in the lower CPA-treated groups, despite the finding of a higher density of medium follicles, which contradicts the studies defending the “burnout theory” where a higher number of follicles initiating activation and growth explain the depletion of the pool [26,28]. Furthermore, we tried to detect the activation of small follicles using the RNAscope assay for *Pten* mRNA, but no obvious signal could accurately be quantified in the oocytes of small follicles in any of the groups. Thus, no evidence of the role of CPA treatment in the small follicles’ activation could be provided in this study. Additionally, assessment of apoptosis also did not demonstrate significant differences in the groups regarding small follicles, not even in the group treated with the highest dose, where a significant reduction of follicles was observed.

For follicle counts on histology, we followed Pedersen and Peters’ classification [18]. Despite a higher number of growing follicles observed in the histological sections, a reduction of about 60% of SeF was observed in the CPA-treated groups. The SeF represents the number of secondary follicles that could be isolated and also fulfilled the morphological conditions for culture. These results confirm results of other studies describing the damage of growing follicles after CPA treatment [3,28]. We should also emphasize that criteria to put in culture, although only based in morphological aspects, are very specific and have been established by extensive research [15,16,17]. In contrast, the group of follicles stained with H&E and counted as growing follicles on histology is heterogeneous [18] and includes follicles that would not be considered for culture. For example, follicles with one layer of granulosa cells are counted as medium growing follicles in the histological analysis, but they do not fulfil the conditions to be put in culture. This may explain the disparity observed between the results obtained in the histological analysis and the number of SeF isolated and cultured. Despite a large reduction in the number of SeF that could be isolated and put in culture from CPA-treated mice, their growth and survival were similar to controls, demonstrating that the criteria of selection to put in culture are efficient and SeF can be rescued after CPA treatment initiation.

PUMA has been described to have an important role in ovarian reserve depletion after cytostatic treatment [29,30,31]. Thus, in this study, we aimed to assess *Puma* mRNA expression in the ovaries treated with CPA. The evidence obtained via RNAscope assay to support the role of *Puma* in follicle damage was weak and the confounding nature of the green autofluoresence encountered does not allow conclusions to be formed. Alternatively, it is also possible that PUMA-independent forms of cell death could be at work, given that PUMA’s role was mainly described in small follicles and the majority of the cell death in this study was observed in the growing follicles.

PTEN is a negative regulator of phosphoinositide 3-kinase (PI3K), and in primordial follicles, it has been described as crucial for the maintenance of follicular dormancy and survival [32]. Thus, in this study we aimed to assess *Pten* mRNA expression after CPA treatment, since some studies have suggested an overactivation of the dormant follicles, leading to an increasing number of growing follicles and “burnout” of ovarian reserve after cancer treatment [26,28]. Interestingly, in this study, the RNAscope assay has demonstrated a clear perifollicular pattern of *Pten* distribution in the growing follicles exposed to CPA, and no or very weak expression of *Pten* mRNA in the small follicles. This manner of localization may indicate that upregulation of *Pten* in thecal cells is a potential marker of follicular damage, or an important aspect of follicular response to CPA exposure. Theca cells are the external layer of growing follicles from the stage of SeF. They are responsible for producing androgens and growth factors that modulate follicular development [16,33,34,35] and their hyperactivity has been associated to infertility [33]. Deletion of *Pten* expression in the theca cells has been demonstrated to lead to androgen excess and ovarian dysfunction in mice [36]. Conversely, PTEN overexpression has been associated with cell cycle arrest and/or apoptosis in several types of cells [37,38,39] and an increased expression of PTEN has been described in the late stages of follicular development in human and ovine species, which could indicate a possible involvement of PTEN in the regulation of granulosa cell proliferation and differentiation [40,41]. Overexpression in theca cells of CPA-treated mice could indicate that the damage in growing follicles will first affect theca cells and by consequence the development and differentiation of granulosa cells. Further studies are required to give a functional evaluation of PTEN overexpression in theca cells. 

In conclusion, while CPA administration produced a significant reduction in the number of viable follicles with good morphologic quality that could be isolated and cultured, our results show that the isolation and culture of SeF in such circumstances was indeed possible. Furthermore, the morphological growth parameters similar to those of isolated follicles from control mice were demonstrated. Despite these promising results, further studies are necessary to evaluate the full normality of the follicles and oocytes such obtained after a chemotherapy treatment, i.e., rates of maturation, meiotic spindle normality, fertilization, developmental competence, and viability of the embryos obtained. If these results prove favorable, the culture of isolated secondary/preantral follicles could be proposed as a fertility preservation alternative for the patients in whom the tissue transplantation is precluded [10,11,42]. Additionally, this study also demonstrated the importance of the theca cells in the response of the growing follicles to damage via upregulated perifollicular *Pten* expression. This is a novel finding whose significance has not yet been fully elucidated.

Our results also indicate a still persistent reserve of small ovarian follicles 3 days after treatment with CPA, especially in the groups that received low CPA doses. These findings support that additional fertility preservation methods, such as ovarian tissue cryopreservation, might be performed even if chemotherapy has been initiated, as the small follicle pool may still be preserved early after the initiation of cancer therapy.

## 4. Materials and Methods

All chemicals used in this study were purchased from Sigma–Aldrich^®^ (Saint Quentin Fallavier, France), unless otherwise indicated.

### 4.1. Animals and Grouping

All animal experimentation described was previously approved by the University Pierre et Marie Curie and by the regional ethics committee for animal research in accordance with the Animal Protection Law, the Animal Protection Regulation, and the Regulation of the Swedish National Board for Laboratory Animals, ID1372 (Date 2012-04-04) and Dnr 1372 (Date 2018-01-24). Experiments were also conducted in accordance with accepted standards of humane animal care.

Twenty peri-pubertal B6CBA/F1 mice (4 weeks old) (Charles River^®^, Lyon, France) were randomly assigned (*n* = 5/group) to each of three groups with CPA treatment: (i) 100 mg/k; (ii) 75 mg/kg; and (iii) 50 mg/kg or to a control group without treatment (iv). CPA, prepared in a sterile 0.9% NaCl solution, was administrated by one single intraperitoneal injection. All mice were sacrificed 72 h after CPA injection by cervical dislocation and the ovaries collected into 37 °C warmed Leibovitz’s L15 media supplemented with 10% fetal calf serum (Gibco^®^, Cergy-Pontoise, France), 100 IU/mL penicillin, and 100 µg/mL streptomycin.

### 4.2. Follicle Isolation and In Vitro Culture

The right ovaries were used for in vitro follicle culture immediately after ovarian collection. Follicles were mechanically isolated under stereomicroscope (Nikon^®^, Champigny sur Marne, France) using 27 gauge needles (Sherwood^®^, Evry, France), ensuring that the follicular structure remained intact and cultured as described by Cortvrindt et al. [15]. Briefly, secondary follicles were selected if they presented healthy morphological characteristics, i.e., having two or more granulosa cell layers with some adhering theca cells, a diameter between 100 µm and 130 µm and a clearly visible round and central oocyte. All follicles were individually cultured in a 20-μL microdrop system, covered by mineral oil at 37 °C and 5% CO_2_ for 12 days [15,17]. An α minimal essential medium with GlutaMAX (α-MEM GlutaMAX, Gibco^®^) supplemented with 5% FBS (Gibco^®^), 10 μg/mL transferrin, 5 μg/mL insulin, and 100 mIU/mL recombinant follicle stimulation (rFSH, GONAL-F, kindly donated from IVF patients) was used. The follicles were daily observed using an inverted microscope (×100) (Nikon^®^) to register morphological characteristics and follicular size. Follicular diameter was estimated using a calibrated ocular micrometer (Nikon^®^) by measuring two perpendicular diameters of the granulosa cell mass without the theca cells. The first day of culture was designated as day 0 and the last day as day 12. Half of the culture medium was renewed from day 2 onwards. At day 12 of culture, follicles with a diameter ≥200 µm, with recognized granulosa cell proliferation and whose oocyte was round and visible, were considered for the endpoint of follicle survival in culture. Additionally, the follicles that reached at least 450 µm of diameter and on which an antrum-like cavity was observed were classified as fully grown follicles.

### 4.3. Histology and Follicle Density

The left ovaries were fixed in paraformaldehyde 4% and kept at 4 °C for 24 h and then transferred to 70% ethanol to be embedded in paraffin. Microtome serial section of 5 μm was performed for each ovary. Every 10th section was H&E stainned, scanned (3Dhistech^®^, Budapest, Hungary), and follicles were counted by two independent observers using Pedersen and Peters’ classification [18] and classified as small, medium, and large. The follicular density was calculated as the total number of follicles divided by the area of ovarian tissue examined (follicle count/mm^2^), as an estimation of the ovarian reserve. Unstained sections were kept for further analysis.

### 4.4. TUNEL Assay

Apoptosis was assessed by TUNEL staining (In Situ Apoptosis Kit (DAB), Abcam^®^, Cambridge, UK). Three sections of each group were deparaffinized, rehydrated, and made permeable by proteinase K. After washing, the sections were labelled with TUNEL reagents according to the manufacturer’s indications (Abcam^®^). Diaminobenzedine (DAB) was used for the development and the counterstaining was made with methyl green. The evaluation was made on scanned sections and the cells were considered positive if they were brown. Positive and negative controls were also performed to confirm efficacy of the TUNEL assay kit and to eliminate any background signal present.

### 4.5. RNAscope for Markers of Follicle Apoptosis and Activation

The mRNA expression of *Puma* and *Pten* were investigated using the RNAscope assay. The probes and the kit were supplied by Advanced Cell Diagnostics (ACDBio, Newark, CA, USA) and applied using a Leica Biosystems BOND-RxM automated stainer (Triolab AB, Mölndal, Sweden). The assay was performed according to the manufacturer’s instructions. A negative control was performed using a probe for a bacterial enzyme (DAP-B) and a positive control using three separate probes to test mRNA quality/degradation in the samples and examine any cross-channel reading interference. The probes were targeted at housekeeping genes *Pol-r2a*, *PpiB*, and *Ubc* channels C1, C2, and C3, respectively. The cells positive to *Puma* were identified with green dots and the ones presenting red dots were considered positives to *Pten*.

### 4.6. Statistical Analysis

All analyses were conducted using SAS^®^, Version 9.4, SAS Institute Inc., Cary, NC, USA. The *t*-test with pooled standard deviation was applied to compare the average follicle diameter, average number of secondary follicles isolated per ovary, and medium follicle density between the CPA treated groups vs. the control group. Survived and fully grown follicle ratios were analyzed using the Cochran-Armitage trend test. *p*-values were adjusted with Bonferroni correction for multiple tests. Differences were considered significant when *p* ≤ 0.05.

## Abbreviations

CPA	cyclophosphamide
H&E	hematoxylin and eosin
Pten	phosphatase and tensin homolog
Puma	p53 upregulated modulator of apoptosis
SeF	secondary follicles
SFD	small follicle density
TUNEL	terminal deoxynucleotidyl transferase dUTP nick end-labelling
